# BIODESERT: Exploring and Exploiting the Microbial Resource of Hot and Cold Deserts

**DOI:** 10.1155/2015/289457

**Published:** 2015-03-26

**Authors:** Ameur Cherif, George Tsiamis, Stéphane Compant, Sara Borin

**Affiliations:** ^1^University of Manouba, Biotechnology and Bio-Geo Resources Valorization (LR11-ES31), Higher Institute for Biotechnology, BiotechPole Sidi Thabet, 2020 Ariana, Tunisia; ^2^Department of Environmental and Natural Resources Management, University of Patras, 2 Seferi Street, 30100 Agrinio, Greece; ^3^Bioresources Unit, Health & Environment Department, AIT Austrian Institute of Technology GmbH, 3430 Tulln, Austria; ^4^Department of Food, Environmental and Nutritional Sciences (DeFENS), University of Milan, Via Celoria 2, 20133 Milan, Italy

Deserts are generally regarded as lifeless and inhospitable ecosystems despite the general awareness of extremophilic microorganisms. An amazing microbial diversity and a huge biotechnological potential were unraveled during the last decade using molecular approaches. Hot and cold deserts were shown to host peculiar microbial assemblages able to cope with hostile environment and/or to rapidly adapt to changing conditions. This adaptation is inferred to particular community structure behavior and specific metabolic capacities allowing cells to overcome water stress, fluctuating temperature, and high salinity. Therefore, such microbes could constitute a source of novel metabolites, biomolecules, and enzymes potentially useful for environmental biotechnologies. With the global climate change, the aridification and creeping desertification that constitute a worldwide serious threat directly affecting agriculture and crop production, and the growing food demands, desert microorganisms could hold the key for green biotechnology and future applications into soil bioreclamation and plant growth promotion for vulnerable regions across the world.

This special issue was focused on the desert microbial resource management (MRM) and how to explore and exploit these resources from hot and cold deserts as well as from arid areas. Aspects of this MRM concept are included in this special issue and they are highlighting (a) the microbial diversity and community structure behavior in desert environments and the identification of novel extremophiles, (b) the influence of the biotic and abiotic factors on microbial communities shape and dynamic and the functional networking including mechanism of adaptation and plant-microbes interaction under extreme or changing conditions, and (c) potential and case applications of desert microbes and/or mixed cultures, such as in soil bioreclamation, reverse-desertification, agriculture, and biomining.

This special issue contains one review and eight research articles that address the three main aspects indicated above. The paper of A. Jaouani et al., entitled “Diversity and Enzymatic Profiling of Halotolerant* Micromycetes* from Sebkha El Melah, a Saharan Salt Flat in Southern Tunisia,” reported the isolation of 21 alkali-halotolerant* Ascomycetes* assigned to the 6 genera* Cladosporium*,* Alternaria*,* Aspergillus*,* Penicillium*,* Ulocladium,* and* Engyodontium*, basing on morphological and molecular markers. Beside their salt and pH tolerance, these saline-system fungi were shown to resist to oxidative stress and low temperature and to produce extremozymes, namely, cellulase, amylase, protease, lipase, and laccase, active in high salt concentrations, which highlight their biotechnological potential. The paper authored by M. del C. Montero-Calasanz et al., “*Geodermatophilus poikilotrophi* sp. nov.: A Multitolerant* Actinomycete* Isolated from Dolomitic Marble,” described a new species within the genus* Geodermatophilus*. Strain G18^T^, isolated from site near the Namib Desert, is characterized by its resistance to heavy metals, metalloids, hydrogen peroxide, desiccation and ionizing, and UV-radiations. Even though 16S rRNA sequence of strain G18^T^ showed 99% similarity with other* Geodermatophilus* species, its taxonomic position and species definition was inferred basing on polyphasic approach and its multitolerance towards environmental stresses, justifying the original given epithet “*poikilotrophi.*”

Four papers were dedicated to the ecological drivers that shape microbial communities and functionalities. A nice example of “cold desert” is presented by S. Ciccazzo et al., “Safe-site Effects on Rhizosphere Bacterial Communities in a High-Altitude Alpine Environment.” In this work, the authors investigated rhizobacterial communities associated with floristic consortia in different safe-sites located in deglaciated terrain. Using DGGE and ARISA, they demonstrated a clear correlation between soil maturation and bacterial diversity and a plant-specific effect leading to the selection of specific rhizobacterial communities by the pioneer plants. Another model ecosystem was investigated by R. Ferjani et al., in the paper “The Date Palm Tree Rhizosphere Is a Niche for Plant Growth Promoting Bacteria in the Oasis Ecosystem.” The work focused on the characterization of the bacterial communities in the soil fractions associated with the root system of date palms cultivated in seven oases in Tunisia using culture-independent and dependent approaches. It was shown that the date palm rhizosphere bacterial communities were rather complex and correlate with geoclimatic and macroecological factors along a north-south aridity transect. However, with the wide diversity of cultivable strains detected, interesting common features of plant growth promoting (PGP) activity and abiotic stress resistance were detected. The authors concluded that palm root system and rhizosphere soil represent a reservoir of PGP bacteria involved in the regulation of plant homeostasis. The third example of plant-microbe interaction is the paper by I. Nouioui et al., in which they demonstrated, as written in the title, the “Absence of Cospeciation between the Uncultured* Frankia* Microsymbionts and the Disjunct Actinorhizal* Coriaria* Species.” The investigation was achieved on five* Coriaria* host species sampled from sites covering the full geographical range of the genus (Morocco, France, New Zealand, Pakistan, Japan, and Mexico). The reported findings argue that* Frankia*, the nitrogen-fixing actinobacteria microsymbionts, have not evolved jointly with their host plants and had probably dispersed globally as a proto-*Frankia*, a free living nonsymbiotic ancestor. The authors hypothesized also that cospeciation may have occurred but subsequently lost after bacteria mixing and fitness selection in the presence of indigenous symbionts.* Frankia* sp. was further investigated in terms of nitrogen fixation under different oxygen tensions. This work authored by F. Ghodhbane-Gtari et al. was conducted on the actinorhizal plant* Casuarina* and its compatible* Frankia* sp. strain CcI3. By studying the growth of the strain, vesicle production, and several genes expression, the authors confirmed the correlation between the biomass and the vesicle production with elevated oxygen tension. It was also shown that oxygen levels influenced nitrogenase induction and that* Frankia* protects nitrogenase by the use of multiple mechanisms including the vesicle-hopanoid barrier and increased respiratory protection. Clearly, the microbial assemblages selected by the plant roots in desert and arid soils are shaped by the ecological biotic and abiotic drivers but with the prerequisite of providing rhizosphere services and specific functionalities.

Biotechnological potential and applications of desert microbes have been reported in three papers. In one, A. Khessairi et al. described a novel efficient pentachlorophenol- (PCP-) degrading halotolerant actinobacterium,* Janibacter* sp. FAS23. The strain was isolated from Sebkha El Naoual, a saline ecosystem in southern Tunisia. Using HPLC analysis, FAS23 was shown to be able to degrade high concentration of PCP (up to 300 mg/l) and to tolerate salt fluctuation. PCP degradation was further enhanced in the presence of glucose and nonionic surfactant tween 80. The strain is considered as a candidate for PCP bioremediation in polluted soils in arid areas. In another paper authored by O. Selama et al., the isolation of haloalkalitolerant and haloalkaliphilic bacteria from Algerian Sahara Desert soil was reported. Thirteen selected isolates, mainly filamentous* Actinobacteria*, were screened phenotypically for antibacterial, antifungal, and enzymatic activities and by PCR for putative antitumor compounds genes. The isolates were assigned to the genera* Streptomyces*,* Nocardiopsis*,* Pseudonocardia*,* Actinopolyspora,* and* Nocardia*, with this latter constituting possibly a new branch in the* Actinomycetales* order. Beside secreted extremozymes and bioactives, several isolates showed antitumorigenic potential. Another paper presented in this special issue is a review article nicely written by A. Azua-Bustos and C. González-Silva, who focused on the Atacama Desert microbes and their current biotechnological applications. A large-scale application in Chile is the copper bioleaching or biomining mediated by indigenous halotolerant and acidophilic chemolithotrophic bacteria like* Acidithiobacillus ferrooxidans* and* Acidithiobacillus thiooxidans*. Other potential applications in arsenic bioremediation and in biomedicine, including the discovery of new antibiotics, antioxidant, antifungal, and immunosuppressive compounds, were cited. The authors reported also application from eukaryotic microorganism as in the case of the halophilic biflagellate unicellular green alga* Dunaliella* that produce beta-carotene.

Definitely, desert environments represent a tremendous reservoir where more efforts, relying not only on metagenomics but also on culturomics, should be dedicated to unravel the hidden potential. The second decade for desert biotechnology has just begun.

